# Combining Stakeholder- and Social Network- Analysis to Improve Regional Nature Conservation: A Case Study from Osnabrück, Germany

**DOI:** 10.1007/s00267-021-01564-w

**Published:** 2021-11-30

**Authors:** Felix Przesdzink, Laura Mae Herzog, Florian Fiebelkorn

**Affiliations:** 1 School of Biology and Chemistry, Didactics of Biology, Osnabrück University, Osnabrück, Germany; 2 School of Cultural Studies and Social Sciences, Institute of Geography and Research Centre Institute of Environmental Systems Research, Osnabrück University, Osnabrück, Germany

**Keywords:** Conservation stakeholders, Regional conservation networks, SWOT analysis, Conservation collaboration, Conservation conflicts, Stakeholder network optimization

## Abstract

Many nature conservation projects fail primarily not because of a lack of knowledge about upcoming threats or viable conservation concepts but rather because of the inability to transfer knowledge into the creation of effective measures. Therefore, an increase in information exchange and collaboration between theory- and practice-oriented conservation actors, as well as between conservation actors, land user groups, and authorities may enhance the effectiveness of conservation goals. By considering the interactions between conservation stakeholders as social networks, social network analysis (SNA) can help identify structural optimization potential in these networks. The present study combines SNA and stakeholder analysis (SA) to assess the interactions between 34 conservation stakeholders in the major city and district of Osnabrück in northwestern Germany and offers insights into cost/benefit optimizations of these stakeholder interactions. Data were acquired using a pile sort technique and guideline-based expert interviews. The SA, based on knowledge mapping and SWOT (strength, weaknesses, opportunities, and threats) analysis, identified individual stakeholder’s complementary properties, indicating which among them would most benefit from mutual information exchange and collaboration. The SNA revealed discrepancies in information exchange and collaboration between theory- and practice-focused stakeholders. Conflicts were found predominantly between conservation associations, authorities and land user groups. Ecological research, funding, land-use conflicts, and distribution of conservation knowledge were identified as fields with high potential for increased information exchange and collaboration. Interviews also showed that the stakeholders themselves see many opportunities for increased networking in the region. The results are discussed in relation to the existing literature on nature conservation networks and used to recommend optimization measures for the studied network. Finally, the conclusion reflects upon the developed approach’s implications and possibilities for conservation stakeholders and planners in general.

## Highlights

- A combination of social network analysis and stakeholder analysis using knowledge mapping and SWOT analysis as a basis for cost/benefit optimization of regional conservation stakeholder interactions.
- A discrepancy in information exchange between theory- and practice-focused conservation actors.
- A high concentration of conflicts between conservation associations, authorities, and land users.
- Intensified networking in the fields of ecological research, land-use conflicts, and conservation knowledge might be prioritized.
- Transfer of the developed method to other regions seems feasible.

## Introduction

The interactions between stakeholders of regional nature conservation can be considered social networks (Bodin et al. 2006; Prell et al. 2009), in which stakeholder interactions influence the effectiveness of regional nature conservation as much as external factors, such as funding or local governmental structures (Bazzoli et al. 2003; Mills et al. 2014; Turrini et al. 2010; Vance-Borland and Holley 2011). Key factors of successful nature conservation and resource management include stakeholder participation (Beierle 2002; Fiorino 1990; Irvin and Stansbury 2004; Kenney et al. 2000; Knight et al. 2006; Renn and Schweizer 2009; Schuett et al. 2001), common stakeholder goals (Cooper et al. 2007; Cornwall 2008; Kenney et al. 2000; Schuett et al. 2001; Williams and Ellefson 1996), jointly coordinating projects (Andonova 2006; Bazzoli et al. 2003; Kenney et al. 2000; Reid et al. 2006), linking theory and practice (Luyet et al. 2012; Schuett et al. 2001; Stern 2005; Williams and Ellefson 1996), and reducing land-use conflicts (Bazzoli et al. 2003; Guerrero et al. 2013). Balmford and Cowling (2006), Guerrero et al. (2013), and Primack (2008) state that an insufficient exchange of interdisciplinary information between theory- and practice-oriented conservation actors as well as a lack of collaboration between land use and conservation stakeholders can lead to ineffective regional conservation. Land-use conflicts (Bazzoli et al. 2003; Germain et al. 2001), inadequate time for cooperative action (Cohen et al. 2012; Korfmacher 2001; Luyet et al. 2012; Williams and Ellefson 1996), and incompatible stakeholder viewpoints (Reed et al. 2009; Williams and Ellefson 1996) are further obstacles of successful conservation projects.

There are many different approaches to “optimize” social networks, for example in terms of resilience, diversity, or particularly short paths between different actors (Thai and Pardalos 2011). Maintaining connections with other stakeholders involves transaction costs, which results in each stakeholder having a maximum threshold of interactions that they can maintain. Therefore, in this study, we focus on optimizing interactions in terms of their cost-benefit efficiency using a combined methodology of social network analysis (SNA) and stakeholder analysis (SA), the latter of which is based on a strength, weaknesses, opportunities, and threats (SWOT) analysis and a knowledge mapping technique. In our network analysis, we strive to identify subsets of actors where the promotion of information exchange and cooperation would be potentially helpful. In addition, as previously recommended by Morgan et al. (2017), we assess current conflicts between stakeholders that could be mediated in the future and identify stakeholders with complementary characteristics that could support each other’s work. Given the small amount of time and resources that many stakeholders can devote to networking and conflict resolution (Berardo and Lubell 2016; Bode et al. 2011; Cohen et al. 2012; Gordon et al. 2013; Luyet et al. 2012), we assume our data collection to be useful for setting up a regional conservation stakeholder database to prioritize potential network interventions and to optimize individual stakeholders’ networking efficiency.

SNA has been used to connect effective regional conservation with structural properties of conservation stakeholder networks (Bodin et al. 2017; Friedman et al. 2020; Guerrero et al. 2013; Mbaru and Barnes 2017; Morgans et al. 2017) to point out gaps in collaboration within such networks (Olsson et al. 2007; Vance-Borland and Holley 2011) and to identify key actors to close these gaps (Cohen et al. 2012; Ernstson et al. 2010). Ernstson et al. (2009), Herzog (2020), Nita et al. (2018), and Vance-Borland and Holley (2011), for example, identified authorities as central actors in regional networks of resource management and nature conservation.

SA is used in natural resource management to obtain information about the stakeholders themselves (Mushove and Vogel 2005), such as assessing their goals or competencies and identifying which stakeholders may function as future key partners for conservation projects (Grimble and Wellard 1997). Some authors view SNA as a useful supplement to SA (Reed et al. 2009), combining, for example, the technique of knowledge mapping with SNA to extend the “who knows who” of SNA with SA data on “who knows what” (Wexler 2001). Such combinations can help prioritize which stakeholders should establish new connections (Prell et al. 2009; Reed et al. 2009) to improve the effectiveness of regional conservation networks (Pressey and Bottrill 2009; Labich 2015; Phillipson et al. 2012). Some studies have already combined SA and SNA into an integrative analytical approach (e.g., Hauck et al. 2016; Lienert et al. 2013), resulting in fruitful methods to study regional environmental management issues.

The present study takes up this approach and expands it by an adapted SWOT analysis (Mintzberg 1994). By analyzing the strengths (S) and weaknesses (W) of stakeholders one can identify their complementarities, the knowledge of which can serve as a base to facilitate networking between those stakeholders who complement each other regarding their resources or knowledge. Furthermore, we analyze the opportunities (O) and threats (T) of more intensive networking from a stakeholders’ perspective and translate the results into recommendations to optimize their social network regarding efficient collaboration while benefitting their resources. Thus, the main objective of this study is to test an innovative multi-method approach combining SNA, knowledge mapping and SWOT analysis, to identify collaboration patterns and complementarities among a network of 34 nature conservation stakeholders in the city and district of Osnabrück, Lower Saxony, in northwestern Germany. The case study of the Osnabrück region combines a medium-sized city (City of Osnabrück with a population of 165,251 and an area of 120 km^2^; Niedersächsisches Landesamt für Statistik 2021), where many stakeholders work in close spatial proximity, with a rural region (District of Osnabrück with a population of 359,471 and an area of 2,100 km^2^; Niedersächsisches Landesamt für Statistik 2021), where actors are physically further apart and their spheres of influence are often limited to the immediate surroundings of their community. In addition, the city has a University as well as a University of Applied Sciences, with a number of academic actors holding knowledge potentially relevant to nature conservation. One reason why there are so many conservation actors in the region is that the city of Osnabrück is located in the midst of several nature reserves (2 Nature Parks, 33 Nature Protection Areas, 23 FFH areas; TERRA.vita 2021). We find here a rather heterogeneous stakeholder landscape including authorities, academics, agriculture, water, fishing, forestry, apiarist, heritage, hunting, and conservation associations. This diverse stakeholder sample seems adequate to develop a methodology that could be used independently of the region.

We draw conclusions about how these stakeholders could optimize the efficiency of their interactions and joint work. In addition, the methods applied to the stakeholder network under study and their transferability to other stakeholder networks in comparable regions are critically reviewed, resulting in recommendations for future studies on the topic. Finally, the conclusion reflects upon the added value of the developed analytical approach for conservation planning in general.

## Material and Methods

As a theoretical basis for the implementation of our multi-method approach, we considered (a) four dimensions of social interactions between the stakeholders, namely awareness among stakeholders, information exchange, collaboration, and conflicts; (b) the availability of specific resources within their network; and (c) the stakeholders’ demands on efficient interactions with other stakeholders. Data analysis and interpretation are guided by the following six analytical steps from SNA and SA, respectively (for a detailed description see the sections “Social Network Analysis” and “Stakeholder Analysis: Knowledge Mapping and SWOT Analysis”):

1. Network density analyses of the four interaction dimensions were conducted to evaluate the intensity of stakeholder interactions. In addition, the densities of information exchange within stakeholders’ common fields of work were analyzed.
2. Community detection algorithms (Girvan–Newman and Walktrap) were used to identify distinct social groups of stakeholders within the four interaction dimensions.
3. Degree and betweenness centrality analyses were used to identify key players who may act as distributors of potential optimization measures inside the stakeholder network.
4. Stakeholders’ self-reported strengths and weaknesses were contrasted to determine whether the weaknesses of some stakeholders could be complemented by the strengths of others.
5. Stakeholders’ perceptions on the opportunities and threats of increased networking were assessed regarding potential future optimization measures.
6. The stakeholder network was interpreted in the context of potential optimization concerning the cost/benefit efficiency of the interactions taking place.

### Stakeholder Identification

This study follows Freeman’s (1984) general definition of stakeholders as “those who affect or are affected by an action,” such as regional nature conservation. While stakeholders can be organizations (Mills et al. 2014) or individuals (Vance-Borland and Holley 2011), for reasons of practicality, only organizations or independently acting sub-organizations were considered in this study. More specifically, we defined stakeholders for this study based on two criteria: (1) organizations that focus most of their work in the city and district of Osnabrück and (2) actively participate in regional conservation projects. Pre-study internet research identified 25 regional authorities, local municipalities, associations, and scientific actors that fit these two criteria. As part of the SA and SNA, the contact person responsible for their regional conservation projects was interviewed for each of these stakeholder organizations. By this, the interactions of each stakeholder organization with the other 24 organizations were surveyed. Furthermore, one wave of snowball sampling (Reed et al. 2009) during each interview was used, asking for additional stakeholders fitting our criteria that were not found through our internet search.

In congruence with Freeman (1984) and Primack (2008), interviewees mentioned different resource user groups as important stakeholders of conservation projects in the region. Thus, regional fishing, agriculture, forestry, water, and hunting associations were added to the stakeholder list. Interviewees also mentioned actors not engaged in or affected by regional conservation but who hold resources relevant for conservation projects (e.g., knowledge on landscape planning). Our initial definition of stakeholders was thus extended by organizations “who could, according to interviewed stakeholders, benefit regional conservation,” resulting in a list of 105 additional stakeholder organizations with the main focus of their work in the city and district of Osnabrück. These additionally mentioned organizations were divided into categories (e.g., “nature conservation association” or “authority”) based on statements from the original 25 interviews. One randomly chosen stakeholder from each stakeholder category that was newly identified was interviewed additionally, leading to 34 interviews in total. Based on the SA, two stakeholders from the provisional list were later assigned to the categories “Water Body Association” and “Heritage Association,” respectively, which is why these categories each contain two stakeholders.

Due to the sampling methodology, it should be noted here that our sample has a slight bias toward organizations actively involved in nature conservation projects with an easy-to-find internet presence. In addition, stakeholder categories identified through snowball sampling are underrepresented. Thus, our sample does not allow drawing reliable conclusions about the complete structure of a potentially larger stakeholder network including all 130 actors. However, since the focus of this paper is to test and discuss the developed methodology, we focus our data analysis and interpretation on this sample.

### Social Network Analysis

A social network models the social system under study (Wasserman and Faust 1994: 93), which in this case includes the interactions between the 34 interviewed stakeholders. It consists of nodes, the stakeholders, and the ties between them, their relations (Wasserman and Faust 1994: 95). Relations between nodes can include, for example, resource or information exchange (Schneider 2014). In this study, SNA is used to assess the status quo of the stakeholder network by analyzing four interaction dimensions: (1) awareness, (2) information exchange, (3) collaboration, and (4) conflict. For each of these four dimensions, network graphs were created using R (R Core Team 2021) and the iGraph Package (v1.2.6; Csardi and Nepusz 2006). Because interviewees may have defined these dimensions differently, the resulting network graphs were treated as directed networks in which not only the existence but also the direction of a tie is relevant (Borgatti et al. 2018). Therefore, stakeholder statements on their interactions with others could be compared with statements of the other stakeholders about their interactions with the one in question.

In more detail, we examined the structure of these network graphs and the positions of single actors within them using the following three measures: (1) communities, (2) network density, and (3) stakeholder centrality.

Communities are subsets of nodes within which node–node connections are dense, but between which connections are less dense (Girvan and Newman 2002). Communities in a social network of stakeholders may thus represent social communities with intense interactions within them but less-intense interactions between them. While many different algorithms for community detection are available, we chose and compared two different approaches for this study. The Girvan–Newman algorithm, also called Edge-Betweenness algorithm, focuses on the edges that are least central in the network and thus most “between” different communities (Newman and Girvan (2004). Edge-betweenness communities are constructed by progressively removing the least central edges from the original graph while measuring the modularity of the respective partition during each step, searching for the partition with the highest modularity. Networks with high modularity have dense connections between the nodes within communities and sparse connections between nodes in different communities (Newman 2006). In contrast to this approach, the Walktrap algorithm uses random walks on the edges of a network to detect communities, as random walks tend to stay into densely connected parts corresponding to communities (Pons and Latapy 2005). The information obtained from conducting large numbers of such random walks is used in a hierarchical clustering algorithm that merges iteratively the vertices into communities. For a more detailed description of the statistics behind both algorithms see Girvan and Newman (2002), Newman and Girvan (2004), and Pons and Latapy (2005).

Network density represents “the proportion of direct ties relative to the possible maximum number” (Wasserman and Faust 1994). We calculated network density for all four interaction dimensions to compare them based on the density of stakeholder interactions. Furthermore, we assessed the network density of information exchange with a specific focus on stakeholder’s common fields of work identified as in the SA to pinpoint fields with low internal densities of information exchange.

Centrality measures focus on the position of single actors within a network (Borgatti et al. 2018: Ch. 10; Wassermann and Faust 1994: 187ff). Because central actors are crucial in diffusing information and influencing the network (Cohen et al. 2012; Ernstson et al. 2010), stakeholders’ degree centrality and betweenness centrality were analyzed to identify such key players. Degree centrality analysis counts the number of ties connecting a specific node with others (Scott 2000). In directed networks, “indegree” centrality measures the number of ties a node receives. In our case, this reflects that the number one stakeholder was being named by other stakeholders as an interaction partner. “Outdegree” centrality measures the number of ties a node sends to others. In our case this reflects the number of interactions with other stakeholders, one stakeholder named (Wasserman and Faust 1994). Stakeholders with high degree centrality values can act as multipliers, disseminating potential optimization measures among many other stakeholders. Betweenness centrality measures the frequency with which one node lays on the shortest path between two others in the network. It sums up the proportion of the shortest paths between all pairs of nodes that pass through the node in focus (Borgatti et al. 2018: Ch. 10). Nodes with a high betweenness centrality may act as “scale crossing brokers,” laying between otherwise unconnected parts of the network (Ernstson et al. 2010). Stakeholders with high betweenness centrality values are of interest, because they could disseminate potential optimization measures between different communities within the network.

### Stakeholder Analysis: Knowledge Mapping and SWOT Analysis

Knowledge mapping creates a visual representation of the distribution of knowledge inside or between organizations (Applehans et al.^1998^; Vail 1999). The structure of a knowledge map varies from actual maps to knowledge databases (Davenport and Prusak 1998). While the technique may represent a useful addition to SA, it has seldom been applied to the context of natural resource management (Reed et al. 2009). This study extends knowledge mapping to a more general mapping of resources: interviews inquired into the stakeholders’ fields of work and their strengths regarding resources the interviewees deemed potentially useful for other stakeholders (e.g., a large pool of volunteers or equipment). Data on common fields of work (e.g., “environmental education” or “forest management”) were interpreted in the context of the network graph of information exchange to compare the densities of information exchange within each field.

SWOT analysis usually focuses on assessing and adjusting the internal behavior, the strengths and weaknesses, of an organization with external factors, the opportunities and threats, of its environment (Kangas et al. 2003; Karppi et al. 2001; Martin-Collado et al. 2013; Mintzberg 1994). These factors are assessed to develop strategies, for example, using strengths to reduce the likelihood of threats or using opportunities to counter weaknesses (Weihrich 1982). Nouri et al. (2008) and Scolozzi et al. (2014) applied SWOT analyses to develop management strategies for protective areas. In this study, the SWOT approach was modified: The stakeholders’ strengths and weaknesses were recorded and compared to assess whether some stakeholder’s strengths may complement other stakeholders’ weaknesses. The record of opportunities and threats focused solely on the context of increased networking between the stakeholders. Because no stakeholders perceived threats resulting from increased networking, threats to increased networking that should be overcome during potential network optimization were recorded instead.

### Data Acquisition: Interviews

To obtain network data, interviewees visualized their organization’s relations to other stakeholders by completing a pile sort task (adapted after Boster 1994 and Boster et al. 1987). They sorted nametags of all stakeholders identified through preliminary internet research into one of five piles: “never heard of” (0), “is known” (1), “information exchange” (2), “collaboration” in a joint project (3), or “conflict” (4). Categories 1–3 were treated as ascending, assuming that the presence of collaboration included mutual awareness and information exchange. Sorting another stakeholder into the category “conflict” could be combined with sorting that same stakeholder into one of the first three categories if both actors have a multi-faceted relationship. The tag positions were translated into network matrices of each interaction dimension. After all interviews had been conducted, a multigrid email questionnaire (Borgatti et al. 2018) was sent to all interviewees. The interviewees sorted their organization’s interactions with stakeholders who were identified by snowball sampling during the data-gathering process into the same categories as they did with stakeholders from the preliminary list in the pile sort task. All interviewees completely answered the questionnaire. The data acquired completed the network matrices, which were analyzed in R (R Core Team 2021) and the iGraph Package (v1.2.6; Csardi and Nepusz 2006). All R scripts can be found in our Open Science Framework repository (https://osf.io/qv3js/).

Stakeholder data were obtained through guideline-based expert interviews (Littig and Menz 2005). The interviewees were asked the following questions, each representing one aspect of the SA: What are your organization’s main fields of work? Where do you see the greatest strengths of your organization, and where could other actors benefit from you? Where do you see weaknesses in your organization, and where could you use support from other actors? Where do you see opportunities for stronger networking among nature conservation stakeholders in the region? What could be the negative effects of additional networking? All interviews took place from October 2019 to March 2020 and were conducted by the first author. The interviews took place on-site at the stakeholders’ organizations headquarters. The mean interview duration was 44 min (SD = 7.7 min). Furthermore, 82% of the interviewees were men and their mean age was 54 years (SD = 13.7 years). In addition, 70% of the interviewees work full time for the stakeholder organization they represent, while 30% are volunteers. The sample is therefore heterogeneous in terms of the type of stakeholder organizations, but narrow in terms of the interviewees’ age and gender. This is not problematic for the interpretation of the results with regard to the case study, as these persons are the representatives of their organizations. In regions with a more diverse demographic background and employment status of contact persons of stakeholder organizations, however, different results would be expected. For a list of the 34 interviewed stakeholders, see the supplementary material. For reasons of anonymity, stakeholder names have been replaced by abbreviations. The interviews were transcribed using Amberscript (2020). Four trained employees of the working group of Didactics of Biology from the Osnabrück University redacted the resulting transcripts following the “easy redaction system” by Dresing and Pehl (2015) and pre-coded them in MAXQDA (VERBI GmbH 2020), assigning each interviewee statement to one interview question and thus to one aspect of the SA. The author performed a qualitative content analysis after Mayring (2010), summarizing and paraphrasing the pre-coded segments into more restrictive codes, which were inductively derived from the interview material. Derived codes included, for example, “ecological research” as a field of work, “need for scientific conservation knowledge” as a stakeholder weakness, or “increased project efficiency” as an opportunity for increased regional networking. Only codes including statements by at least two interviewees were included in the SA. These codes are shown in Table 2 (fields of work), Table 3 (strengths and weaknesses) and Fig. 2 (opportunities and threats). For the complete coding tree, see the supplementary material.

## Results

### Density Analysis and Community Detection

The results of the density analysis and community detection are shown in Table 1.

**Table 1:** Descriptive statistics and community detection results of the four interaction dimensions awareness (upper left), information exchange (upper right), collaboration (lower left), and conflict (lower right)

Network dimension	Awareness	Information exchange
No. of nodes	34	34
No. of ties	862	462
Density	77%	43%
Avg. degree centrality	25.3	14.4
Edge-betweenness communities		
		
Walktrap communities		
		
Network dimension	Collaboration	Conflicts
No. of nodes	34	34
No. of ties	351	40
Density	35%	3.6%
Avg. degree centrality	10.6	1.1
Edge-betweenness communities		
		
Walktrap communities		
		

In each case, the upper network graph represents the results of an edge-betweenness community detection, while the lower network graph represents the results of a walktrap community detection. Nodes belonging to the same community are identically colored and surrounded by the same colored shape. Due to a restricted amount of different colors, nodes or shapes from different communities may have identical colors, but for each community the combination of node color and shape color is unique.

*AUT* authority, *FOU* foundation, *MUN* municipality, *CON* conservation association, *HUN* hunting association, *WAT* water body association, *FIS* fishing association, *AGR* agriculture association, *FOR* forestry association, *API* apiarist association, *HER* heritage society, *UNI* university working group, *UNA* University of Applied Sciences working group

The network graph on mutual awareness scores a very high density of 77%, indicating that most stakeholders are familiar with each other. Both community detection algorithms detect one large community (red) that excludes the university working groups that represent their own single-actor communities (edge-betweenness model) or one common community (walktrap model).The density value decreases to 43% when information exchange is examined. Thus, significantly fewer stakeholders exchange information than are aware of each other. In this network, edge-betweenness again sorts most actors into one common community (red), while almost all academic actors, a heritage association, and an apiarist association form their own single-actor communities. Walktrap asserts the university working groups to one community (green) and detects two more communities comprising the University of Applied Sciences working groups together with some conservation associations and authorities (blue) and the majority of other stakeholders (red). The collaboration network scores a density value of 35%, meaning 71% of the stakeholders who exchange information collaborate on a project as well. Edge-betweenness gives the exact same result as in the information exchange network. Walktrap creates one community of the university actors (blue). The University of Applied Sciences actors are grouped together with some conservation associations, the heritage associations, and apiarist association (red), while the rest of the network is again plotted in one large community (green). In the network of conflicts, the density value is at 3.6% significantly lower. For both algorithms, the stakeholders who did not mention conflicts at all are plotted as single-actor communities. Both algorithms detect slightly different communities between the few stakeholders who are engaged in conflict. More importantly, the examination of the individual edges shows that 27% of all conflicts occur between land user groups and authorities, 24% between land user groups and conservation associations, and 21% between conservation associations and authorities.

Overall, the results indicate that with regard to information exchange, collaboration academic- and most practice-oriented stakeholders are grouped into separate communities with the university working groups also being outside of the main community regarding awareness. Conflicts occur between few land use actors, authorities, and conservation associations, while most scientific actors did not report any conflicts. Conflicts exist between 35% of the stakeholders and this group can be regarded as highly conflictual with 25% of all possible conflictive ties between these stakeholders present.

### Information Exchange in Common Fields of Work

The SA identified 11 fields of work that were common to several stakeholders (Table 2). The most common ones were environmental education (18 stakeholders), compensation measures (17 stakeholders), and grassland management (16 stakeholders). The allocation of stakeholders to the individual fields of work is shown in Table 2.

**Table 2:** Allocation of stakeholders to common fields of work

Field of work (FoW)	WN	EE	CM	GM	NW	SM	WM	FM	MP	FD	GV	ER
No. of stakeholders in FoW	34	18	17	16	15	15	15	11	11	9	9	9
Density of inf. exch. in FoW [%]	43.0	38.2	59.5	52.1	61.9	50.9	54.8	70.0	56.4	59.7	81.9	45.8
Normalized density [%]	77.0	38.2	56.7	46.2	51.7	39.6	45.8	42.7	34.2	30.0	41.0	23.0
Stakeholder category	Percentage of stakeholders active in respective field of work											
Authorities	100	15	86	71	71	57	57	71	71	71	86	15
Foundations	100	0	0	0	100	0	0	0	0	100	0	0
Municipalities	100	100	100	100	66	66	100	100	0	0	100	0
Conservation association	100	15	43	57	43	57	28	15	28	0	0	15
Hunting association	100	100	0	100	0	100	0	100	0	0	0	0
Water association	100	0	100	0	50	0	100	0	0	50	0	0
Fishing association	100	0	0	0	0	100	100	0	0	0	0	100
Agriculture association	100	0	100	100	100	0	0	0	0	0	0	0
Forestry association	100	0	100	0	0	100	0	100	0	0	0	0
Apiarist association	100	0	0	0	0	0	0	0	0	0	0	0
Heritage association	100	0	0	0	100	0	0	0	0	50	0	0
University working group	100	100	0	25	0	25	50	0	50	0	0	75
Univ. of. Appl. Scien. working group	100	100	33	33	0	66	33	0	66	33	0	100

The percentages in the cells represent the proportion of stakeholders in that stakeholder category who are active in that field of work. For each field of work, the density and normalized density of the information exchange network between active stakeholders are given. Values were normalized in relation to the field of work indicated by most stakeholders, environmental education.

*WN* whole network, *EE* environmental education, *CM* compensation measures, *GM* grassland management, *NW* networking, *SM* species management, *WM* water management, *FM* forest management, *MP* mapping, *FD* funding, *GV* governance, *ER* ecological research

The following fields of work score the highest normalized internal densities of information exchange: compensation measures (56.7%), networking (51.7%), grassland management (46.2%), and water management (45.8%). Stakeholders working in the same field exchange less information with each other than they do within the entire network of stakeholders (normalized density of 77%). The lowest internal densities of information exchange are found in funding (30%) and ecological research (23%).

### Central Stakeholders or “Key Players”

**Fig. 1:**
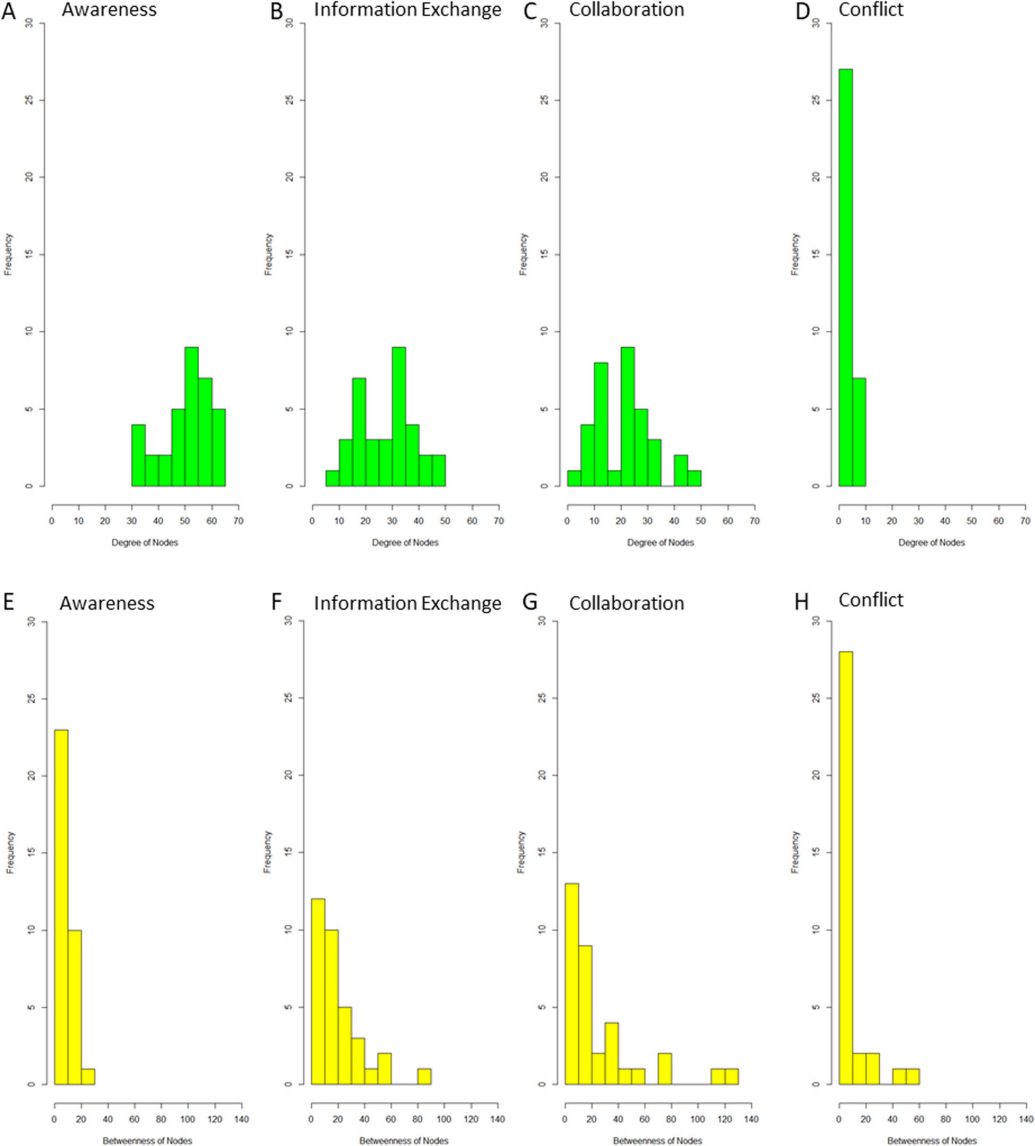
Results of degree centrality (**A**–**D**) and betweenness centrality (**E**–**H**) analyses. Each chart shows the number of stakeholders (frequency, *y*-axis) scoring-specific centrality values (*x*-axis)

The distribution of node degrees initially reflects the network density of the four dimensions of social interaction, awareness, information exchange, collaboration, and conflict (Fig. 1A–D). In the awareness network (density of 77%), all stakeholders have a degree centrality above 30 (Fig. 1A), while 17 stakeholders in the information exchange network (density of 43%; Fig. 1B) and only 6 in the collaboration network (density of 35%; Fig. 1C) also score a degree value above 30. In the collaboration network, six of the ten stakeholders that have the highest outdegrees are also among the top ten stakeholders regarding indegree (Supplementary Table 2). These include three state authorities (AUT3, AUT4, and AUT5), one foundation (FOU1), one conservation association (CON10), and one water association (WAT3). In the information exchange network, AUT3, AUT4, FOU1, and CON10 are again among the ten actors with the highest indegree and outdegree (Supplementary Table 2). These four stakeholders are thus the most active and central players in the networks of positive interactions. Considering the conflict network (Fig. 1D), the large proportion of actors (22) not involved in conflicts has no incoming or outgoing ties and thus a degree centrality of zero. Seven stakeholders from the land use sector (forestry association FOR2 and agriculture association AGR1), the state (AUT3 and AUT4), and the conservation sector (CON1, CON2, and CON3) have relatively high degree measures ranging between six and ten (Supplementary Table 2) as can also be seen in Fig. 1D. These stakeholders are involved in the majority of conflicts in the network.

Regarding betweenness centrality measures, all actors in the highly dense awareness network have low betweenness centralities with a measure of maximum 25 (Fig. 1E). In the information exchange network, one single actor (the authority AUT3) has a betweenness centrality of almost 80 with a large gap toward the values of the other stakeholders (Fig. 1F). This indicates that this stakeholder is by far best able to efficiently pass information between actors in the network. In the collaboration network, two authorities (AUT3 and AUT4) have betweenness values above 100. These two authorities therefore play an important role as scale crossing brokers in this network. The state authorities AUT3 and AUT4, the foundation FOU1 and the nature conservation association CON10 are among the highest-scoring stakeholders in the centrality analyses on information exchange and collaboration and thus may act as central actors and scale crossing brokers simultaneously.

### SWOT Analysis: Complementary Strengths and Weaknesses

The assessment of stakeholder strengths and weaknesses summarized in Table 3 showed seven common weaknesses. The most frequently mentioned are involvement in land-use conflicts (11 mentions), insufficient practical conservation knowledge (10 mentions), and funding issues (7 mentions). Seven common strengths were identified, with practical conservation knowledge (19 mentions), possession of areas suitable for conservation projects (10 mentions), funding expertise and scientific conservation knowledge (9 mentions each) noted most frequently. For all identified weaknesses, stakeholders with complementary strengths exist in the network (Table 3). For only one weakness, involvement in land-use conflicts, the number of stakeholders mentioning it (11) surpasses the number of stakeholders mentioning a strength (4) that could complement this weakness. In the other cases, the number of “strong” stakeholders equals or surpasses the number of “weak” stakeholders regarding the specific topic.

**Table 3:** Results of the stakeholders’ strengths and weaknesses analysis (*n* = 34)

Stakeholder category	Land-use conflicts		Practical conserv. knowledge		Funding		Scientific conserv. knowledge		Public image		Areas suitable for conserv. projects		Machinery	
	Involved	Mediates	Needs	Has	Problems	Expertise	Needs	Has	Negative	Good	Needs	Has	Needs	Has
Authorities	43%	14%	28%	85%	0%	71%	14%	43%	14%	43%	0%	14%	0%	14%
Foundations	0%	0%	0%	0%	0%	100%	0%	0%	0%	0%	0%	0%	0%	0%
Municipalities	0%	0%	0%	100%	0%	0%	0%	0%	0%	0%	0%	100%	0%	0%
Conserv. association	71%	28%	0%	71%	43%	0%	43%	28%	0%	14%	43%	14%	43%	0%
Hunting association	0%	0%	100%	100%	0%	0%	0%	0%	100%	0%	0%	0%	0%	100%
Water association	50%	0%	50%	0%	100%	50%	0%	0%	0%	50%	0%	100%	0%	50%
Fishing association	100%	0%	100%	0%	100%	0%	0%	0%	100%	0%	0%	100%	0%	0%
Agriculture association	100%	100%	0%	100%	0%	100%	0%	0%	100%	0%	0%	100%	0%	0%
Forestry association	100%	0%	100%	0%	100%	0%	0%	0%	100%	0%	0%	100%	0%	0%
Apiarist association	0%	0%	0%	0%	0%	0%	0%	0%	0%	0%	0%	0%	0%	0%
Heritage association	0%	0%	50%	0%	0%	50%	0%	0%	0%	0%	0%	0%	0%	0%
University working group	25%	25%	50%	75%	0%	0%	50%	25%	0%	0%	0%	0%	0%	0%
Univ. of Appl. Scien. working group	0%	0%	33%	33%	0%	0%	0%	100%	0%	0%	0%	0%	0%	0%
Total number of stakeholders	11	4	10	19	7	9	6	9	5	5	3	10	3	3

The first line shows superordinate topics in which weaknesses and complementary strengths were grouped. Below, weaknesses are shown in the left column and strengths in the right column of each topic. The percentages in the cells represent the proportion of stakeholders in each category who indicated the respective strength or weakness. The last line shows the total number of stakeholders mentioning each specific weakness or strength

Only authorities, conservation associations and most land user groups are involved in land-use conflicts. Scientific or practical conservation knowledge exists throughout all stakeholder categories except for foundations and most land user groups. Funding problems were mentioned only by conservation associations and land user groups. Scientific conservation knowledge is only possessed by four of seven scientific actors in the network, but also by some authorities and conservation associations. Only conservation associations are in need of machinery and areas suitable for conservation projects, both of which can be offered almost exclusively by actors from the governance and land use sectors.

### SWOT Analysis: Opportunities and Threats

**Fig. 2:**
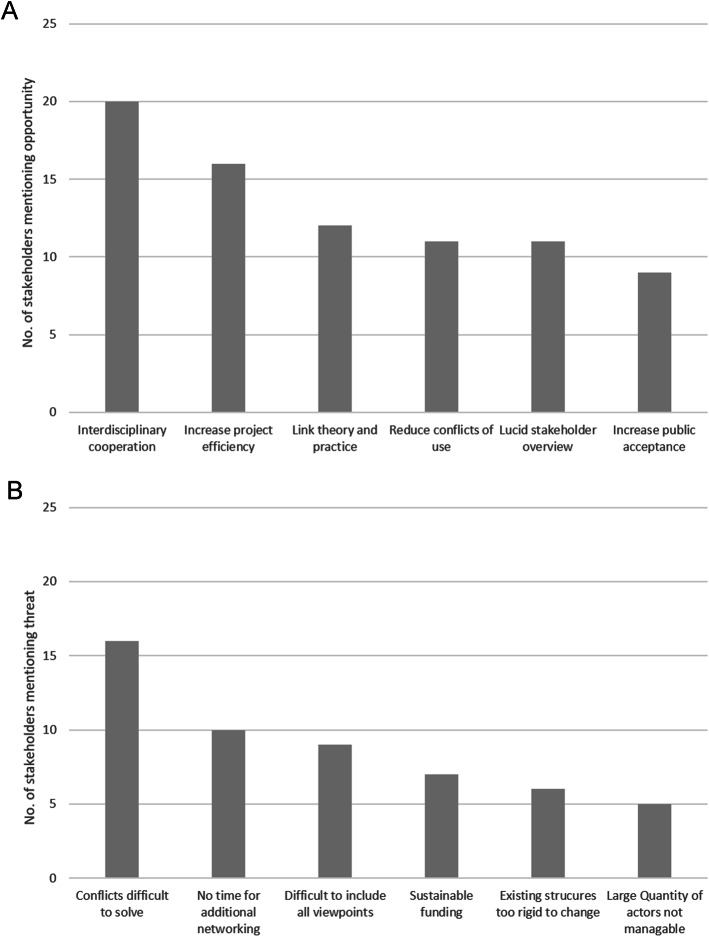
Results of the opportunities and threats analysis (*n* = 34). **A** Opportunities of increased networking with other stakeholders regarding conservation issues as mentioned by the stakeholders. **B** Threats to increased networking with other stakeholders regarding conservation issues as mentioned by the stakeholders

Stakeholders’ statements on opportunities of increased regional networking on conservation issues were summarized to six superordinate opportunities shown in Fig. 2A. Higher levels of interdisciplinary collaboration (20 mentions) and increased project efficiency (16 mentions) were the highest-ranking categories. None of the stakeholders reported threats of increased networking on conservation issues with other stakeholders, but all of them reported threats to such ambitions. These have been sorted into six superordinate threats shown in Fig. 2B. Unsolvable conflicts between stubborn or emotional “hardliner” stakeholders (16 mentions) and the short amount of time stakeholders could spend on additional networking (10 mentions) were the highest-ranking threats.

## Discussion

### Network Structure

The examined networks show high network densities. We assume that this can be attributed to the narrow limitation of our sample to the nature conservation sector of a rather small region. The decreasing network density from awareness to information exchange to collaboration reflects the fact that these interaction dimensions were treated as additive during data acquisition, assuming that collaboration includes information exchange, which in turn includes awareness. The distribution of communities in the information exchange and collaboration networks shows a discrepancy between academic- (e.g., University and University of Applied Sciences actors) and practice-focused (e.g., conservation associations and authorities) stakeholders, reflecting a “science practice gap” (Bertuol‐Garcia et al. 2018; Fabian et al. 2019). Most conflicts exist between the stakeholder categories “authorities,” “conservation associations,” and “land use actors.” These results show a lack of interdisciplinary information exchange between theory- and practice-focused stakeholders as well as a lack of collaboration between land use, governance, and conservation actors, even if all are critical for successful regional conservation (Balmford and Cowling 2006; Guerrero et al. 2013; Primack 2008).

The high density of the entire networks of positive interactionsis also reflected in the different fields of work. The fact that all fields of work have lower densities of information exchange than the information exchange network as a whole may seem counterintuitive, as networking with others in one’s own field appears to make more sense than networking with actors from different fields. However, as an actor from the field of work “funding” stated, “We network primarily with actors who need our money and not with those who have money to lend themselves.” In this case, networking outside one’s own field of work may indeed make more sense than networking within it. This point is taken up again in the study’s “Limitations and Transferability” section.

### SWOT Analysis

The most frequently mentioned stakeholder weaknesses are involvement in land-use conflicts, insufficient practical conservation knowledge, and funding issues. These weaknesses correspond to typical problems conservation projects have (Guerrero et al. 2013; Knight et al. 2006; Labich 2015; Primack 2008). For each of the weaknesses stakeholders mentioned, other stakeholders with complementary strengths exist, as far as the rough definition of “strengths” and “weaknesses” allows. This definition is scrutinized in more detail in the “Limitations and Transferability” section. This finding underlines the potential for a collaborative, supportive co-creation of future conservation projects among the stakeholders, which may also lead to an overall increased information exchange and collaboration. This potential coincides with the assessment of stakeholder’s views on the opportunities and threats that increased regional networking may have on conservation issues. Here stakeholders view possible opportunities as more important than possible threats and all stakeholders named at least one opportunity, while none mentioned a threat. The SNA data do reflect this result in that densities of awareness, information exchange, and collaboration between the sampled stakeholders are high. Thus, stakeholders seem to appreciate working closely together. However, the fact that stakeholders did not mention any threats of increased networking may be caused by a confirmation bias or a social-desirability bias on side of the actors, or a “core bias” during the stakeholder identification. This point is further discussed in the “Limitations and Transferability” section. The opportunities mentioned by the stakeholders largely correspond to the most important factors for successful nature conservation projects: interdisciplinary collaboration has been reported in the form of stakeholder participation (Beierle 2002; Fiorino 1990; Irvin and Stansbury, 2004; Kenney et al. 2000; Knight et al., 2006; Renn and Schweizer 2009; Schuett et al., 2001), common stakeholder goals (Cooper et al. 2007; Cornwall 2008; Kenney et al. 2000; Schuett et al. 2001; Williams and Ellefson 1996), and the joint coordination of projects (Andonova 2006; Bazzoli et al. 2003; Kenney et al., 2000; Reid et al. 2006). Numerous authors also note that increased project efficiency, whether through improved financing (Kenney et al. 2000; Labich 2015; Schuett et al. 2001), increased political influence (Kenney et al. 2000; Labich 2015; Lampe and Kaplan 1999), or interdisciplinary collaboration itself, is crucial. Furthermore, researchers have pointed to the importance of linking theory and practice (Luyet et al. 2012; Schuett et al. 2001; Stern 2005; Williams and Ellefson 1996) and reducing land-use conflicts (Bazzoli et al. 2003; Guerrero et al. 2013). Factors that stakeholders perceive as threats to increased networking appear in the literature as inhibitors of functioning stakeholder collaboration: unresolvable personal conflicts (Bazzoli et al. 2003; Bodin et al. 2020; Germain et al. 2001), a lack of time for additional cooperative action (Cohen et al. 2012; Korfmacher 2001; Luyet et al. 2012; Williams and Ellefson 1996), incompatible viewpoints (Reed et al. 2009; Williams and Ellefson 1996), and insufficient funding (Bazzoli et al. 2003; Cohen et al. 2012; Mostert 2003) must be overcome to improve regional conservation efforts.

### Optimization Potential in the Analyzed Network

As the density analysis suggests, the present stakeholder interactions are already in a very dense state regarding awareness, information exchange, and collaboration. However, interdisciplinary information exchange between theory- and practice-focused actors should be emphasized. Since information exchange takes place to a lesser extent in all shared fields of work than in the information exchange network itself, a special focus should be given to fields of work with low densities of information exchange, such as ecological research. In future interviews with the stakeholders, one could ask which fields of work would actually benefit from stronger internal networking and which ones are more dependent on networking with stakeholders from other areas of work (e.g., possibly the field of funding). Additional community detections within each field of work may be able to further specify which stakeholder groups would benefit from increased information exchange.

The network-level results of the SNA can be contextualized with stakeholder-level results from the SWOT analysis. The combined interpretation of both datasets shows that in the case of “scientific conservation knowledge,” strengths are distributed throughout theory- and practice-focused actors, while communication occurs more frequently within these groups than between them, making additional between-community information exchange a valuable option here. The same holds true for collaboration between conservation and land use actors and the distribution of strengths and weaknesses between them regarding resources. Special emphasis should be placed on tackling land-use conflicts since, in this context, the number of stakeholders needing assistance far outweighs the number of stakeholders offering it. Furthermore, land user groups should be included in the distribution of conservation knowledge. Stakeholders with high betweenness centralities may play crucial roles in facilitating exchange between communities and in involving peripheral areas, while stakeholders with a high degree centrality may be helpful for quickly implementing specific measures for large proportions of the network. Since the two authorities AUT3 and AUT4, the foundation FOU1, and the nature conservation association CON10 scored high centrality values in all indices, these organizations may become key partners for network optimization measures. The identified opportunities should be emphasized, and solutions for the identified threats should be communicated to activate regional stakeholders for network optimization.

**Fig. 3:**
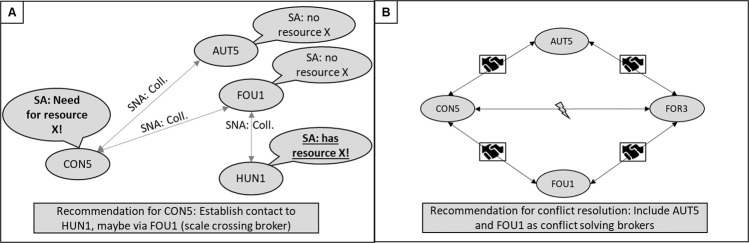
Using the combination of SNA/SA for stakeholder consultation. **A** A simplified network graph symbolizing the use of SNA and SA results for networking recommendations on the individual level. SA results (speech bubbles) show that conservation association CON5 needs a resource that hunting association HUN1 has. SNA results (arrows) show that they do not collaborate with each other (Coll.: collaboration). A possible recommendation for **A** is listed below. **B** The combined collaboration ego network of conservation association CON5 and forestry association FOR3, who are in conflict with each other (lightning). The network graph is reduced to nodes that collaborate (shaking hands) with CON5 and FOR3. These could be of use as “conflict solving brokers,” based on the concept of scale crossing brokers

In addition to such recommendations at the network level, the results of the regional SA should be made available to local stakeholders. If they need support in certain areas or are looking for partners for a new project, the information on knowledge and resources of other stakeholders may be helpful in finding optimal cooperation partners. For instance, the need for a resource by one stakeholder could be satisfied by establishing a collaboration with another stakeholder who possesses this resource and perhaps including a common collaboration partner as a scale crossing broker (Ernstson et al. 2010), as depicted in Fig. 3A. A conflict between two stakeholders could be overcome more easily by including joint partners as mediators or “conflict solving brokers” (Fig. 3B).

This approach simplistically assumes that recommendations made based on SNA and SA are considered and implemented by the participating stakeholders on a cost-benefit basis. In practice, of course, interactions between stakeholders are also significantly influenced by psychological (e.g., mutual trust, attitudes, emotions) and political (e.g., pre-set agendas or alliances) factors (Cohen et al. 2012; Morgans et al. 2017; Williams and Ellefson 1996). This should be considered when implementing network interventions, and the mere provision of information to stakeholders may need to be complemented by more elaborate interventions to build shared trust, reduce conflict, or create shared visions (Luyet et al. 2012; Morgans et al. 2017; Reed 2009). Establishing platforms, such as policy forums, through which stakeholders can engage in negotiations, discussions, joint projects, and social learning (Fischer and Leifeld 2015) could be an advantageous and sophisticated way to foster stakeholder collaboration.

## Limitations and Transferability

Several potential limitations in this study should be considered. Interviewees may have distorted some results by giving preference to actors sympathetic to them or by exaggerating or understating the strengths and weaknesses of their own organization. The study first included stakeholders with a strong online presence in the initial internet research. As the interviews made clear, smaller, locally active players rarely have a strong internet presence. However, these stakeholders in particular are likely to have few ties only and could represent a large part of the peripheral network. Also, during snowball sampling stakeholders who were well known to the interviewees and presumably had better network connections were probably mentioned first, while small peripheral stakeholders were only mentioned in passing. Consequently, the sample of the 34 stakeholders examined in this study could exhibit a bias toward the core of the bigger regional network of conservation stakeholders. In terms of the data presented here, this would indicate that the high network densities found in the sample may not be representative of the whole network of conservation stakeholders in the Osnabrück region.

The coarse definitions for what constitute a strength or a weakness make a confirmation bias possible. For example, a “strong” actor may not have exactly the “scientific conservation knowledge” that a “weak” actor needs, but a complement is indicated nonetheless. Here, a detailed discussion of the results with the individual “strong” and “weak” stakeholders would be important to determine the individual cases in which they actually “match.” The same holds true for the perceived opportunities of increased regional networking, which value concrete positive results of an optimized network on the same level as “common sense” statements such as “working together with other disciplines is always good.” No stakeholder made any statements regarding the threats of additional networking. However, regarding threats to additional networking projects, stakeholders mentioned aspects that could be interpreted as threats of increased networking (e.g., too little time for stakeholders to invest in networking). This could be due to a social-desirability bias that led interview partners to underestimate the dangers of additional networking “for the sake of the study.” In order to investigate what constitutes a “good” network, one has to consider the context in which the network under investigation is located and to perform more elaborate network analyses than just basic descriptive network statistics. As Turrini et al. (2010) pointed out, network effectiveness relies on a variety of aspects such as the “capacity of achieving stated goals,” “sustainability,” or “innovation,” both at the community level and at the level of individual actors. Consequently, an examination of the different network densities and the SWOT analysis results can only serve as a starting point in determining a network’s optimization potential. Joint discussions with stakeholders could clarify, for example, whether a more intensive exchange of information within specific fields of work with low network densities would increase efficiency from the stakeholders’ point of view or whether less networking would be required in these areas. The SNA and SWOT linkage, which is still rudimentary in this study, could also be deepened by using a two-mode network analysis (Borgatti et al. 2018), thereby studying interactions between “strong” and “weak” actors to determine whether complementarity fails due to a lack of mutual awareness, a lack of information exchange, or the presence of conflicts.

This study’s results and conclusions are specific to the studied sample. SNAs in other regions may reveal different factors as most important to increase interaction efficiency in the respective networks. If, for example, a stakeholder network in a rural region shows different villages forming single communities or even components (stakeholder groups that are completely separated from each other; Borgatti et al. 2018), the starting point for network optimization may be to connect these villages instead of addressing phenomena highlighted in this study. At the same time, the methodological approach used in this study is readily transferable to other regions, as no prior knowledge of the actors and the network under study is necessary. Practitioners elsewhere may thus use it to identify specific areas in their regional nature conservation network to either intensify stakeholder interaction or encourage the resolution of conflicts. “Stakeholder databases” based on regional SNAs and SAs could also be set up in many regions and, if sufficient resources are available, accompanied by a “network consulting” to facilitate the most efficient networking possible for local stakeholders. As stated in the last section, such ambitions may need to be supplemented by more elaborate interventions, depending on the stakeholders’ willingness to cooperate with each other.

## Conclusion

This study’s analytical approach combined an SNA of the interactions between 34 nature conservation stakeholders from the region of Osnabrück, Germany, with an assessment of their common fields of work and complementary strengths and weaknesses through knowledge mapping and SWOT analysis. The SNA identified discrepancies in information exchange between theory- and practice-focused stakeholders and between conservation associations and a cluster of authorities and resource user groups, with the latter three also involved in most of the network’s conflicts. Fields of work with low internal densities of information exchange can act as initial starting points for facilitating more intensive networking. However, such prioritization should also inquire about and consider the perceived need for intensive networking by stakeholders in the individual fields. While the SNA indicated high densities of mutual awareness, information exchange, and collaboration in the stakeholder network, the SWOT analysis revealed complementary strengths and weaknesses between many actors. Two authorities, one foundation and one conservation association were identified as the central actors in the network and thus as key players who could disseminate recommendations in the stakeholder network. However, due to the data collection through interviews, the results of the SNA and SWOT analysis could be subject to a core bias as well as a confirmation and a social-desirability bias; a potential short-coming most studies relying on interview data face.

Despite the study’s limitations, the authors give concrete recommendations to improve the investigated stakeholder network regarding compensation for some stakeholder’s weaknesses by others’ strengths and an increase of information exchange between disciplines and across specific fields of work. Our multi-method approach should be applied to the larger stakeholder network in the Osnabrück region and improved through further case study applications. Results of these analyses should then be made accessible for the regional stakeholders. Further fostering of networking activities through “network consulting” by conducting SNAs and SAs on a regular basis could be a desirable option to improve regional stakeholder interactions in the long term. These consultants may develop measures to solve problems identified by analyzing the whole network and consult individual stakeholders on how best to optimize their own networks or overcome existing conflicts.

## Supplementary information

Coding tree

Suppl. Tab. 1.1

Suppl. Tab. 1.2

Suppl. Tab. 1.3

Suppl. Tab. 2

Suppl. Tab. 3
